# Estimation of the Intrinsic Power Efficiency in Magnetoelectric Laminates Using Temperature Measurements

**DOI:** 10.3390/s20113332

**Published:** 2020-06-11

**Authors:** Xin Zhuang, Chung-Ming Leung, Jiefang Li, Dwight Viehland

**Affiliations:** Department of Materials Science and Engineering, Virginia Tech, Blacksburg, VA 24061, USA; cmleung@vt.edu (C.-M.L.); jili4@vt.edu (J.L.); dviehlan@vt.edu (D.V.)

**Keywords:** magnetoelectric effect, power efficiency, equivalent circuit model, magnetoelectric gyrator

## Abstract

Magnetoelectric (ME) power efficiency is a more important property than the ME voltage or the current coefficients for power conversion applications. This paper introduces an analytical model that describes the relation between the external magnetic field and the power efficiency in layered ME composites. It is a two-phase model. The first fragment establishes the expression between the magnetic field strength and the temperature increase within an operating period. It uses a magneto-elasto-electric equivalent circuit model that was developed by Dong et al. Following previous investigations; the main loss source is the mechanical power dissipation. The second fragment links the power efficiency and the temperature increase in a heat-balanced system. This method is generally used by researchers in the piezoelectric field. The analytical model and the experimental data shows that the decrease of the power efficiency in a laminated composite is between 5% and 10% for a power density of 10 W/in^3^ (0.61 W/cm^3^) to 30 W/in^3^ (1.83 W/cm^3^). The failure mechanism/process of ME composites under high power density can be estimated/monitored by the proposed method for ME composites in practical applications.

## 1. Introduction

The intrinsic power efficiency of a magnetoelectric (ME) composite is causally related to the power loss and the coupled power in the magnetic-to-electric conversion process. Under an oscillation drive, a power loss is present during the energy conversion per cycle. Accumulated energy dissipation results in an increase of the ambient temperature, like in piezoelectric actuators, in a ME composite [1]. By consequence, the power loss and power transmission characteristics can be estimated by monitoring the rising of the temperature [2,3,4,5]. Consequently, it is possible to estimate the intrinsic power efficiency by monitoring temperature. A ME laminated composite often consists of one piezoelectric and two magnetostrictive layers, which are bonded together using epoxy or sintering techniques [6,7]. ME composites with numerous structures and materials have been wildly investigated as magnetic field sensors and power conversion devices [8,9], although it is still an open question to their potential benefits and limitations with regards to their applications as next-generation devices [10,11].

To characterize the ME effects in a massively layered composite, there are two main approaching methods based on constitutive equations: the continuum mechanics model (CMM) that was introduced by Bichurin et al. and the equivalent circuit model (ECM) that is introduced by Dong et al. The CMM treats a ME layered composite as a continuum object that is fulfilled with the magnetostrictive, piezoelectric materials and the intermediate layer [12]. With this model, the conservation of the energy can be independently investigated in different phases. The coupling loss at the intermediate layer can be readily described by the CMM. Instead, the ECM lumps together the magnetostrictive and piezoelectric phases as a magneto-elasto-electric equivalent circuit, where the mechanical strain/stress within the two phases are not outward parameters.

The ECM has a concise expression that allows engineers to draw an equivalent circuit for a ME composite. However, when the measurements are not in accordance with the theoretical analysis, the loss is not able to be clearly identified. In this case, it needs to introduce a correction factor with a general loss mechanism including the eddy current (Foucault current), boundary condition, mechanical loss or flux concentration. This cannot guide engineers towards a clear target to improve the ME composite in practical applications because the physical meaning of the correction factor is too general. Actually, the used coefficients in layered ME composites are “predefined” values for the magnetostrictive and piezoelectric materials, rather than their effective values in a specific ME composite. In particular cases, it is much more operational to measure the effective coefficients than correction factors.

Effective coefficients in composite materials are different from their individual participants, which was discussed by Newnham and Cross in their work *Piezoelectric Ceramic-Polymer Composites for Transducer Applications*. The content that involved a bulk-bulk connectivity was developed by Zhuang et al. which took into account a sandwich structure of one Pb(Zr,Ti)O_3_/polymer layer surrounded by two solid Pb(Zr,Ti)O_3_ surface layers [13], where the composite coefficients showed their effective values. However, it was still a difficult task to demonstrate the effective coefficients in composite materials because of the amount of input coefficients needed to identify their effective values. Therefore, it would be wise to simplify the expression into a figure of merit (FOM) with a simple and refined formula before exploring their effective values. For the performance of the piezoelectric transducers, Safari proposed a product of the piezoelectric charge and voltage coefficients, *d_p_ g_p_* for his thesis at Pennsylvania State University [14]. Later, Bhalla et al. proposed another FOM which took into account not only the piezoelectric properties, but also the dielectric loss tangent *d_p_ g_p_/tanδ* [15]. For the layered ME composites, the ME voltage, current coefficients and dissipation in ECM expressions were demonstrated as a FOM with a product of the squared coupling factor and the mechanical quality factor (*k*^2^*Q*) to evaluate the power conversion efficiency by Zhuang et al. [16]. Subsequently, by using the measured effective coefficients as inputs for this simplified FOM, the simulation was carried out and compared to the measurements. The results showed that the ECM with effective coefficients can predict the measurement with high accuracy for layered ME composites [17].

Inaccurate coefficients in a model might result in important errors to the final prediction. Besides the mentioned method—simplified FOM with effective inputs—the measurement of one or several intermediate values was another way to monitor the error propagations before a huge accumulation occurred in the model. In a layered ME composite, the intermediate values could be the magnetostriction, the vibration velocity or the temperature increase. In this manuscript, we established a theoretical relation between the intrinsic power efficiency in a laminated ME composite and the applied magnetic field strength with the temperature increase as the intermediate value to monitor the functioning of ME composites.

## 2. Heat Energy System

**Figure 1:**
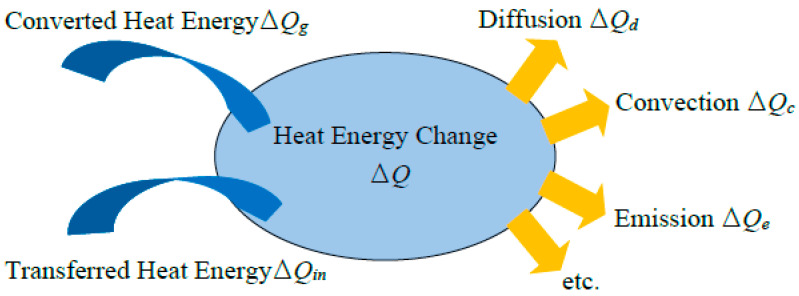
Sketched view of a heat balance system.

The temperature rising, Δ*T*, in a ME laminate is due to the heat energy change, Δ*Q*, which can be expressed as an energy balance equation. For a thermal balance system, the heat energy increase is due to the input heat energy, *Q_in_*, and the generated energy, *Q_g_*, from other types of energy, meanwhile the decrease is due to the diffusion *Q_dif_*, the convection *Q_c_*, the emission *Q_e_* and the energy conversion to other types of energy. The dynamic thermal balance is conceptually illustrated in Figure 1.

The sum of all the heat energy results in a change of temperature in the ME system. The temperature change related to the heat energy balance can be calculated by taking into account the mass and the mean specific heat capacity of the system, yielding

(1) $$Q_{g}+Q_{in}+Q_{dif}+Q_{c}+Q_{e}+\ldots =\Delta Q=c_{p}m\Delta T,$$

where *c_p_* is the mean specific heat capacity, *m* the mass and Δ*T* is the temperature change.

Without input heat energy, an increase of heat energy in a mechanically coupled ME system is mainly due to a dissipation process, such as the dielectric loss in the piezoelectric phase, the eddy current (Foucault current) loss in the magnetic phase and the mechanical loss in both phases. It can be assumed that all the dissipated energy is converted into heat energy with time. Then, we obtain an equality between the dissipated energy (*Q_d_*), and the dissipated power (*P_d_*) with time, given as:

(2) $$Q_{g}=Q_{d}=P_{d}\Delta t.$$

The input heat energy *Q_in_* is the heat energy flow into the system from a heat source. In the case for a ME sample, we assume that there is not a heat source near the sample (i.e., *Q_in_* = 0). Brownian motion of the gas molecules can result in heat exchange between the ME sample and the air, which can be expressed as *Q_dif_*. For a driven ME sample, the heat energy radiates from the ME sample to the air, the transmission is expressed via Fourier’s law,

(3) $$q_{dif}=\kappa \left(T_{ME}-T_{air}\right)=\kappa \Delta T,$$

where *q_dif_* is the thermal transfer power per thermo-contact surface area, *κ* is the thermal transferring coefficient, *T_ME_* and *T_air_* are the temperatures in the ME sample and in the air. Based on this equation, the diffused heat energy can be expressed as

(4) $$Q_{dif}=-q_{dif}S_{urface}\Delta t=-\kappa S_{surface}\Delta T\Delta t,$$

where *S_surface_* is the thermo-contact surface and Δ*T* is the temperature difference during a period of the time (Δ*t*) for the energy diffusion. The heat energy change due to the air convection is governed by Newton’s cooling law, given as

(5) $$q_{c}=h\left(T_{ME}-T_{air}\right)=h\Delta T,$$

where *h* is the heat convection coefficient. The heat energy change due to the convection within a period of time can then be written as

(6) $$Q_{c}=-q_{c}S_{urface}\Delta t=-hS_{urface}\Delta T\Delta t.$$

Thermal radiation emission reduces the heat energy, which is expressed by the emission equation, yielding

(7) $$q_{e}=\varepsilon_{m}\sigma \left(T_{ME}^{4}-T_{env}^{4}\right),$$

where *ε_m_* and *σ* are the emissivity and Stefan–Boltzmann constant, and *T_env_* is the environmental temperature. Thus, we have the heat energy change due to the emission, yielding

(8) $$Q_{e}=-q_{e}S_{urface}\Delta t=-\varepsilon_{m}\sigma S_{urface}\left(T_{ME}^{4}-T_{env}^{4}\right)\Delta t.$$

The heat energy change resulting from emission is small in a ME laminate composite. Hence, it can be assumed that the term *Q_e_* is neglectable. The equality of the thermal balance system in a magnetically driven ME laminated composite can then simplified as

(9) $$Q_{g}+Q_{dif}+Q_{c}=c_{p}m\Delta T.$$

## 3. Dissipation Induced Heat Energy

The heat energy resulting from the mechanical dissipation can be expressed as a product of the power dissipation (*P_d_*) and a period of the time. This expected power loss can be calculated from the velocity (root mean square value, rms) and the real part of the mechanical impedance, given as

(10) $$Q_{d}=P_{d}\Delta t=v_{rms}^{2}Re\left\{Z_{m}\right\},$$

where *v_rms_* is the rms velocity and *Re*{*Z_m_*} is the real part of the impedance. The relation between the velocity and an applied magnetic field (*H*) can then be established via the mechanical impedance, yielding

(11) $$j\omega \varphi_{m}H\left(\omega \right)=v\left(\omega \right)Z_{m}=v\left(\omega \right)\left(\frac{1}{j\omega C_{m}}+R_{m}+j\omega L_{m}\right),$$

where *C_m_*, *R_m_* and *L_m_* are the mechanical capacitance, resistance and inductance respectively. *φ_m_* is the magneto-elastic coupling. For a ME laminate driven at its resonant frequency (*ω_res_*), the term $\frac{1}{j\omega_{res}C_{m}}+j\omega_{res}L_{m}$ is zero. The rms amplitude of the vibration velocity is proportional to the strength of the excitation magnetic field, given as

(12) $$\nu \left(\omega_{res}\right)=\frac{\omega_{res}\varphi_{m}H\left(\omega_{res}\right)}{R_{m}}.$$

By using Equations (10)–(12), the heat energy change due to the mechanical dissipation can be derived as

(13) $$\begin{array}{ll} Q_{d} & =P_{d}\Delta t=v_{rms}^{2}Re\left\{Z_{m}\right\}\Delta t\\ & =\frac{1}{2}{\left(v\left(\omega_{res}\right)\right)}^{2}Re\left\{Z_{m}\right\}\Delta t=\frac{\omega_{res}^{2}\varphi_{m}^{2}{\left(H\left(\omega_{res}\right)\right)}^{2}}{2R_{m}^{}}\Delta t \end{array}$$

## 4. The Temperature Rising Modeling

By taking into account the assumption that the generated energy results from mechanical dissipation, we have *Q_g_* = *Q_d_*. Thus, the thermal balance equations can be rewritten from Equations (4), (6), (9) and (10) as

(14) $$\begin{array}{l} Q_{d}+Q_{dif}+Q_{c}=c_{p}m\Delta T\\ \int P_{d}dt-\int \kappa S_{surface}\Delta Tdt-\int hS_{urface}\Delta Tdt=c_{p}m\Delta T \end{array}$$

From Equation (14), we have the temperature increase as a function of the driving time,

(15) $$\Delta T=\frac{1}{2R_{m}^{}}\frac{\omega_{res}^{2}\varphi_{m}^{2}{\left(H\left(\omega_{res}\right)\right)}^{2}}{\left(\kappa +h\right)S_{urface}+\frac{c_{p}m}{\Delta t}}.$$

By taking the initial temperature *T*_0_, after a period of the driving time (Δ*t*), the finial temperature *T_fA_* can be given as

(16) $$T_{fA}=T_{0}+\frac{1}{2R_{m}^{}}\frac{\omega_{res}^{2}\varphi_{m}^{2}{\left(H\left(\omega_{res}\right)\right)}^{2}}{\frac{c_{p}m}{\Delta t}+\left(\kappa +h\right)S_{urface}}.$$

The resonant frequency and the mechanical resistance are $\omega_{res}=1/\sqrt{L_{m}C_{m}}$ and $R_{m}=\omega_{res}L_{m}/Q_{mech}$, respectively. Here, *L_m_*, *C_m_* and *Q_mech_* are mechanical inductance, capacitance and quality factor. Thus, Equation (16) becomes

(17) $$T_{fA}=T_{0}+\Delta T=T_{0}+\frac{1}{2L_{m}}\frac{\omega_{res}\varphi_{m}^{2}{\left(H\left(\omega_{res}\right)\right)}^{2}Q_{mech}}{\frac{c_{p}m}{\Delta t}+\left(\kappa +h\right)S_{urface}}.$$

A look-up table is given in Table 1, which consists of the mentioned parameters and their definitions. The magneto-elastic coupling is $\varphi_{m}=d_{33,m}A_{m}/s_{33}^{H}=d_{33,m}nA/s_{33}^{H}$, where *d*_33,*m*_ is the effective magnetostrictive coefficient under certain magnetic bias, *A* is the intersection area of the sample and *s^H^*_33_ is the compliance of the Metglas used. The mechanical inductance is $L_{m}=\frac{1}{\pi^{2}}\left(m_{MG}+m_{PZT}+m_{g}\right)$, where *m_MG_*, *m_PZT_* and *m_g_* are the mass of the Metglas, PZT and epoxy, respectively. *R_t_* is the thickness percentage of the epoxy. 1/*R_t_* defines the ratio of the thickness of the epoxy to the total thickness of the sample. ρ_MG_, ρ_PZT_ and ρg are the volume density for the Metglas, PZT and the epoxy, respectively. *V_MG_*, *V_PZT_* and *V_g_* are the volume values for Metglas, PZT and epoxy, respectively. The mechanical capacitance is $C_{m}=\frac{sl}{A}=\frac{l/\left(wt\right)}{\frac{n}{s_{33}^{H}}\left(1-\frac{1}{Rt}\right)+\frac{\left(1-n\right)}{s_{11}^{E}}\left(1-\frac{1}{Rt}\right)+\frac{1}{s_{g}Rt}}$, where *s^H^*_33_, *s^E^*_33_ and *s_g_* are the compliance values for Metglas, PZT and epoxy, respectively. *l*, *w* and *t* are the length, width and thickness of the sample. The thermo-contact surface is calculated from the sample geometry, yielding $S_{urface}=2\left(lw+wt+lt\right)$. The power loss, *P_loss_* is the difference between the input and output power, thus it can be calculated as

(18) $$P_{loss}=\frac{PD\times Vol}{\eta }\times \left(1-\eta \right),$$

where *η* is the power efficiency, *PD* is the power density and *V_ol_* is the volume of the ME composite. The temperature rising can be calculated from the power loss as well, by inserting Equation (18) into (14). It yields

(19) $$T_{fB}=T_{0}+\Delta T=T_{0}+\frac{\frac{\left(1-\eta \right)}{\eta }\times PD\times Vol}{\frac{c_{p}m}{\Delta t}+\left(\kappa +h\right)S_{urface}}.$$

## 5. Results and Discussion

A Metglas PZT laminate, consisting of a PZT plate (30 mm × 6 mm × 0.3 mm) that was sandwiched by two Metglas layers, was fabricated using a hot pressing technique in a vacuum bag. Each Metglas layer had six foil thin films bonded together with epoxy. Additionally, PZT and Metglas were bonded together with epoxy at 60 °C. A bobbin solenoid with a 300-turn number winding coil fed this ME laminate by a magnetic field. An optimal resistive load was connected to the PZT layer. The energy that was converted by the ME laminate per cycle can be calculated from the power coupled on the load resistor and the volume of the ME laminate. For power densities of 10 W/in^3^ and 30 W/in^3^, the output power levels measured from the load resistor were 0.06 W and 0.182 W, respectively.

**Figure 2:**
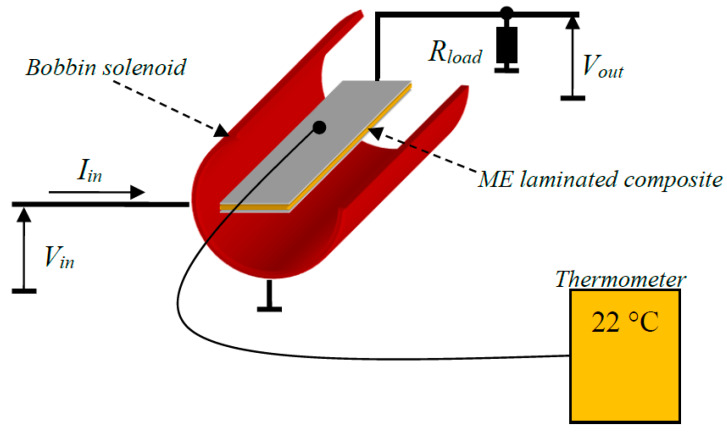
Experimental set-up for the temperature measurement.

The temperature was measured by means of a thermal couple. The probe of the thermal couple was placed into the coil and close to the ME laminate, as shown in Figure 2.

**Figure 3:**
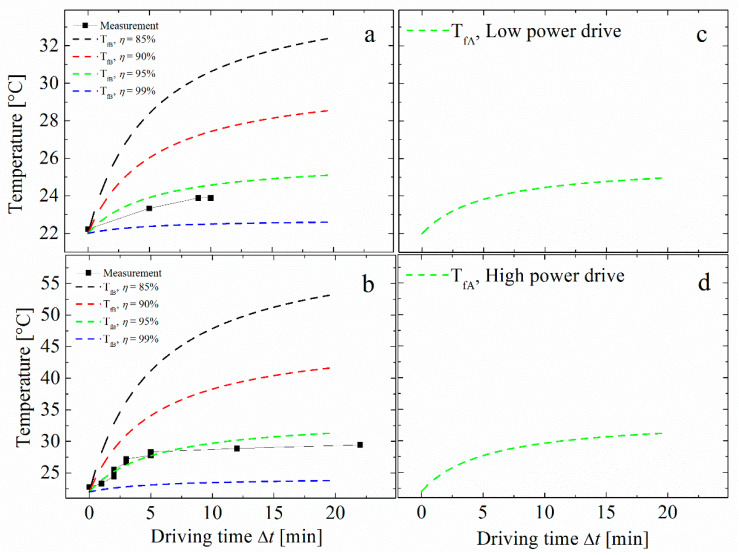
Temperature rising as a function of the driving time with a power density of (**a**) 10 W/in^3^ and (**b**) 30 W/in^3^. The dashed lines are simulations with Equation (19) and the black squares are experimental data. Simulations using Equation (17) under low drive and high drive conditions are shown in (**c**,**d**), respectively.

Under a power density of 10 W/in^3^, the measured temperature increased from 22 °C to 24 °C within 10 min, as shown in Figure 3a. Using Equation (19), the calculated temperature increase in a ME laminate with power efficiencies of 85%, 90%, 95% and 99% are shown as dashed black, red, green and blue lines respectively. Due to a huge heat convection in the coil, it is assumed that the temperature rise can mainly be attributed to the power dissipated by the ME laminate. Thus, our results show that the intrinsic power efficiency of ME laminates can expect to be higher than 90%. Retaining an optimal resistive load, the power density was increased up to 30 W/in^3^. The temperature then increased from 22 °C to near 30 °C after a drive time of more than 20 min, as shown in Figure 3b. Using a finger to touch the ME laminate after driving, we did not feel any obvious change in temperature as well. Using Equation (17), simulated results are given in Figure 3c,d. These results are consistent with the measurements and calculated values using Equation (19), as given in Figure 3a,b. The parameters used in Equation (17) are given in Table 2.

As an intermediate parameter, the temperature can be linked to the applied magnetic field via the ECM in Equation (17). Thus, the coefficients in the model can be monitored by comparing the simulation and measurements in Figure 3a,b. For example, the model can return a temperature difference when a decrease of the mechanical quality factor occurred. Meanwhile, the temperature can also evaluate and calibrate the power efficiency with Equation (19) when the ME device is operated under high power condition.

## 6. Conclusions

By using an equivalent circuit model, the temperature rise in magnetoelectric composites was established as a function of an external magnetic field. Considering the variation in heat energy, this temperature rise can also be calculated from the power loss and the power density of a ME laminated composite. Compared with the measured data, the intrinsic power efficiency of this ME laminate is estimated. When the ME composite was connected to a load resistor with an optimal value, the measurement revealed that the intrinsic power loss percentage was estimated as a value lower than 10% while the ME laminate was driven under a power density up to 30 W/in^3^ (1.83 W/cm^3^). A measured temperature can help to adjust the coefficient in the model to minimize the error propagation, such as the decrease of the quality factor. This temperature can also monitor the power efficient variation that is resulted by the load change at users’ ends.

## Figures and Tables

**Table 1:** Parameter look-up table.

Parameter	Symbol	Parameter	Symbol	Parameter	Symbol
Initial temperature	*T*_0_ (K)	Magnetic field	*H* (A/m)	Thermal conductivity coefficient	*κ* (W/(m^2^ K))
Angular frequency	*ω* (rad)	Effective mass	*m* (kg)	Heat convection coefficient	*h* (W/(m^2^ K))
Magneto-elastic coupling	*φ_m_* (N/(A/m))	Mean specific heat capacity	*c_p_* (J/(kg K))	Thermal contact surface	*S_urface_* (m^2^)
Mechanical quality	*Q_mech_*	Driving time	Δ*t* (s)	Mechanical inductance	*L_m_* (kg)

**Table 2:** Values of the parameters for the simulations.

Parameter	Value	Parameter	Value
Compliance of Metglas, *s^H^*_33_ (m^2^/N)	9.1 × 10^−12^	Length, *l* (m)	30 × 10^−3^
Magnetostrictive coefficient, *d*_33,*m*_ (m/A)	650 × 10^−12^	Width, *w* (m)	6 × 10^−3^
Mechanical quality factor, *Q_m_*	100	Thickness, *t* (m)	0.522 × 10^−3^
Volume density of Metglas, *ρ_m_* (kg/m^3^)	7290	Thickness ratio, *n*	0.456
Volume density of PZT, *ρ_p_* (kg/m^3^)	7700	Epoxy thickness fraction, *R_t_*	100
Volume density of epoxy, *ρ_g_* (kg/m^3^)	1097	Initial temperature, *T*_0_ (K)	295
Mean specific heat, *c_p_* (J/(kg K))	350	Driving time, Δ*T* (min)	20
Thermal conductivity of air, *κ_air_* (W/(m^2^ K))	0.024	Magnetic field strength @ 10 (30) W/in^3^, *H* (Oe)	0.085 (0.15)
Convection factor, *h* (W/(m^2^ K))	2.0	Resonant angular frequency, *ω_res_* (rad)	350 × 10^3^
